# Noncoding RNA in Oncogenesis: A New Era of Identifying Key Players

**DOI:** 10.3390/ijms140918319

**Published:** 2013-09-05

**Authors:** Guorui Deng, Guangchao Sui

**Affiliations:** 1 Department of Cancer Biology and Comprehensive Cancer Center, Wake Forest University School of Medicine, Winston-Salem, NC 27157, USA; 2 Hypertension and Vascular Research Center, Wake Forest University School of Medicine, Winston-Salem, NC 27157, USA; E-Mail: gdeng@wakehealth.edu

**Keywords:** noncoding RNA, microRNA, gene expression, cancer, tumor suppressor, oncogene

## Abstract

New discoveries and accelerating progresses in the field of noncoding RNAs (ncRNAs) continuously challenges our deep-rooted doctrines in biology and sometimes our imagination. A growing body of evidence indicates that ncRNAs are important players in oncogenesis. While a stunning list of ncRNAs has been discovered, only a small portion of them has been examined for their biological activities and very few have been characterized for the molecular mechanisms of their action. To date, ncRNAs have been shown to regulate a wide range of biological processes, including chromatin remodeling, gene transcription, mRNA translation and protein function. Dysregulation of ncRNAs contributes to the pathogenesis of a variety of cancers and aberrant ncRNA expression has a high potential to be prognostic in some cancers. Thus, a new cancer research era has begun to identify novel key players of ncRNAs in oncogenesis. In this review, we will first discuss the function and regulation of miRNAs, especially focusing on the interplay between miRNAs and several key cancer genes, including p53, PTEN and c-Myc. We will then summarize the research of long ncRNAs (lncRNAs) in cancers. In this part, we will discuss the lncRNAs in four categories based on their activities, including regulating gene expression, acting as miRNA decoys, mediating mRNA translation, and modulating protein activities. At the end, we will also discuss recently unraveled activities of circular RNAs (circRNAs).

## 1. Introduction

The central dogma of biology dictates that genetic information flows in a unidirectional fashion of DNA-mRNA-proteins. Thus, the majority of the early research in molecular and cellular biology was focused on the protein-coding genes and their transcripts, messenger RNAs (mRNAs). However, in the past two decades, noncoding RNAs (ncRNAs) have garnered increased appreciation for their important roles in regulating various biological processes. In the whole human genome, more than 90% of the DNA sequence can be transcribed, but only about 2% of it encodes proteins [1–5]. Recent studies reveal that most of these excess transcripts are not transcriptional noise, but rather serve as functional ncRNAs regulating chromatin modifications, gene transcription, mRNA translation and protein function [5–7].

The discovery of the first human oncogene RAS [8–10] and the first tumor suppressor Retinoblastoma (Rb) in the 1980s invigorated the field of cancer research [11,12]. The fact that a simple point mutation in the RAS gene could transform a normal growth-regulating gene into a cancer-causing factor inspired cancer researchers to seek out additional cancer-regulating genes. In the decades that followed, the search for novel oncogenes and tumor suppressors has dominated the field of cancer research, leading to the discovery of many well-characterized tumor suppressors, such as p53, and oncogenes, such as c-Myc. With the discovery of ncRNAs and their involvement in oncogenesis, a new surge of research in determining the oncogenic and tumor suppressive roles of these noncoding transcripts has taken place. To date, multiple classes of ncRNAs have been demonstrated to regulate different biological or physiological processes. It is possible that some ncRNAs may not have a discernible function; however, the enormous number of the identified noncoding transcripts and their differential expression profiles in cancers indicate that we are still at the beginning of this exciting era to explore the functions and regulatory mechanisms of ncRNAs in cancers.

NcRNAs can be divided into three categories based on length or number of nucleotides (nts) [13]. (1) Short ncRNAs: these RNAs are 17–30 nts in length and include microRNAs (miRNAs), piwi-interacting RNAs (piRNAs), and transcription initiation RNAs (tiRNAs). MiRNAs will be one of the major focuses of this review; (2) Middle-size ncRNAs: these RNAs have variable sizes but are typically between 20 and 200 nts in length. The small nucleolar RNAs (snoRNAs) belong to this category; (3) Long ncRNAs (lncRNAs): these RNAs are over 200 nts. This category includes several well-characterized ncRNA, such as MALAT1 and HOTAIR. Circular RNAs (circRNAs) have recently been discovered and can be grouped here based on their sizes [14]. Many members in this category were identified in the last few years, which will be extensively discussed in this review.

Due to rapid expanding of the ncRNA field, it is difficult to cover all aspects of ncRNA-related studies in a single review article. There are many excellent reviews discussing the activities of ncRNAs from different perspectives. In this review, we will discuss the biological function of miRNAs and lncRNAs in cancers, with particular attention paid to their interplay with p53, c-Myc and PTEN. We will also summarize recent studies of circRNAs that have returned to the arena with a recently unraveled function since their discovery many years ago.

## 2. MicroRNAs

### 2.1. Introduction of MicroRNAs

**Figure 1:**
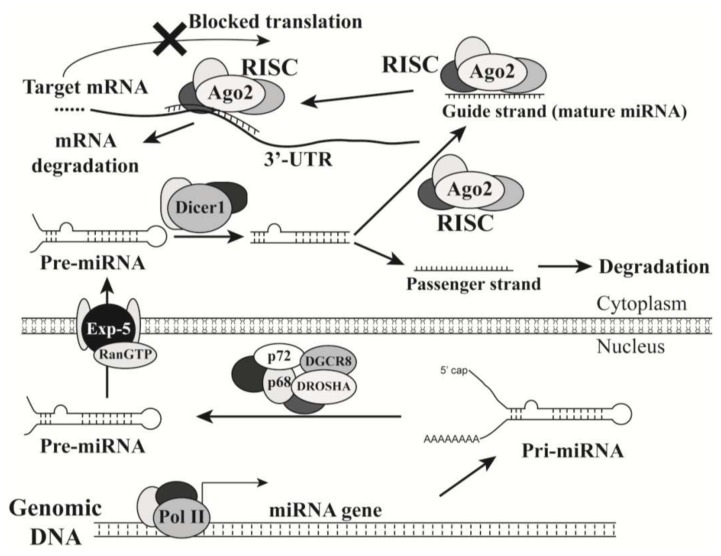
MicroRNA biogenesis and their role in inhibiting gene expression. A miRNA is transcribed by RNA polymerase II (Pol II) mediated by other transcription factors, such as p53 and c-Myc. The generated pri-miRNA is processed by the DROSHA complex to become a pre-miRNA, which is transported from nucleus to cytoplasm with a complex consisting of Exportin-5 (Exp-5) and Ran-GTP. In cytoplasm, the pre-miRNA is further processed by a Dicer-1 complex to become a duplex that consists of a guide strand (to become a mature miRNA) and a passenger strand. The guide strand associates with the RNA-induced silencing complex (RISC) and guides it to the target site on the 3′-UTR of an mRNA to inhibit the mRNA translation and cause mRNA degradation. For some miRNA-duplexes post Dicer-1 processing, both strands can become mature miRNAs; the one at the 5′-end of pre-miRNA is suffixed by “-5p”, while the 3′-end one is suffixed by “-3”, such as miR-17-5p and miR-17-3p.

MiRNAs are small and evolutionarily conserved noncoding RNAs that are typically 18 to 25 nts in length. The biogenesis of miRNAs begins with the synthesis of the primary transcripts (pri-miRNAs) by RNA polymerase II in the nucleus (Figure 1). Like the transcripts of protein-coding genes, pri-miRNAs contain a 5′ cap structure, a poly(A) tail, and sometimes intron sequences [15,16]. A stem loop structure can be formed by regions of partial complementary sequences in each pri-miRNA. The nuclear ribonuclease DROSHA and its partner DGCR8 recognize this stem-loop structure, and subsequently crop the pri-miRNA to generate the pre-miRNA intermediate. After the pre-miRNA is exported to the cytoplasm by Exportin-5/Ran-GTP, it is further processed by DICER1, another ribonuclease, to generate a double-stranded miRNA molecule. Both strands can act as mature miRNAs; however, if only one strand (guide strand) becomes a functional miRNA, the other strand (passenger strand) will be quickly degraded [17,18].

A mature miRNA can associate with Argonaute proteins to form a RNA-induced silencing complex (RISC), in which the miRNA guides the complex to the 3′-untranslated region (3′-UTR) of the target mRNA to block translational protein synthesis and/or cause its degradation (Figure 1). Each miRNA contains a seed sequence (7 nts; from the nucleotides 2 to 8) at its 5′-end with conserved complementarity to perfectly pair the ‘seed-match’ sequence at the 3′-UTR of its target mRNA [19,20]. Since only a 7-nucleotide match between a target 3′-UTR and a miRNA seed sequence will theoretically make the mRNA a potential target of the miRNA, miRNAs do not have a high specificity for their targets like small interference RNAs (siRNAs) have. MiRNAs can be divided into different families. A miRNA family consists miRNAs that share the same seed sequence and thus may target the same set of genes [19]. MiRNA coding regions, or their genes, can be located in either protein-coding or noncoding regions of transcription units in the human genome. Each of these regions may encode one or a cluster of mRNAs. A miRNA cluster is a set of two or more miRNAs that are transcribed from physically adjacent miRNA genes. Thus, the miRNAs in a cluster are transcribed by the same promoter, in the same direction, and as an unseparated transcription unit. One cluster typically contains two or three miRNAs, but large clusters do exist, such as the miRNA-17-92 cluster consisting of seven miRNAs. Interestingly, some members in one miRNA family are encoded by one miRNA gene cluster, such as miR-15a/miR-16-1 and miR-34b/miR-34c. Different miRNA clusters may belong to the same miRNA family, such as miR-15a/miR-16-1 and miR-497/miR-195.

To date, over 2,500 human microRNAs have been identified based on the microRNA database (miRBase) at the Sanger Institute (http://www.mirbase.org/). Bioinformatical analyses suggest that miRNAs may regulate over 5,300 human genes, which represents 30% of human genes [19] and each miRNA regulates about 200 genes [21]. Thus, altered expression of microRNAs in cancer can cause significant perturbation of gene expression with a profound effect on malignant transformation and cancer progression [20].

There are much debate and investigation regarding the mechanisms of miRNA-repressed protein expression. Initially, miRNA-mediated gene silencing was portrayed as a different mechanism from that of siRNA-mediated mRNA degradation, based on the observation that the lin-4 miRNA decreased lin-14 protein expression without affecting its mRNA levels in *C. elegans* [22,23]. This was supported by multiple studies showing that miRNA-mediated gene silencing could be achieved with no or minor change of the target mRNA levels in *Drosophila* [24], *Arabidopsis* [25] and mammalian cells [26,27]. However, several later studies suggested that microRNAs can both block translation and trigger target mRNA degradation [28,29]. Notably, a recent report by Djuranovic *et al.* provided new insights into the kinetics of miRNA-mediated gene silencing in *Drosophila* cells. The authors demonstrated that miR-9b and miR-279 first repress the translation of the target mRNAs and then cause mRNA deadenylation and degradation [30]. Whether this mechanism is applicable to all miRNAs or other species remains to be determined.

Through the ability to repress the expression of multiple genes, miRNAs play an important role in regulating many cellular activities such as proliferation, differentiation and apoptosis. Calin *et al.* provided the evidence showing the deletion of the miR-15/miR-16 cluster at 13q14 and its downregulation in patients with B cell chronic lymphocytic leukemia [31]. This was the first study suggesting that noncoding genes correlate with and may even contribute to oncogenesis. The same group further investigated the loci of 186 miRNAs in the human genome to evaluate their potential involvement in cancer pathogenesis [32]. They discovered that over 50% of these miRNA genes are present in the genomic regions with reported alterations in cancers. These are highly instable loci, including fragile sites, minimal heterozygous deletion regions, frequently amplified sections and common breakpoints. However, the instability of these miRNA-coding regions does not generate frequent miRNA somatic mutations within their seed sequences in cancers but rather changes their expression. A more recent study indicated that the genes encoding oncogenic miRNAs are mainly located in the amplified regions in human cancers, whereas the majority of genes for tumor suppressive miRNAs are in the deleted regions [33]. Interestingly, many oncogenes can produce alternative mRNA isoforms with shorter 3′-UTR sequences through a mechanism involved in alternative cleavage and polyadenylation [34]. A short mRNA isoform of an oncogene can avoid miRNA-mediated inhibition, which consequently increases its stability and typically produces ten-fold more protein. Overall, miRNA expression is globally reduced in tumors compared to their matched normal tissues [35]. Thus, numerous studies have demonstrated the potential of using the expression profiles of single or multiple miRNAs as biomarkers to classify tumor origins, stages and clinical outcomes [36–39]. Currently, microRNA expression detection is not used clinically in cancer diagnosis and prognosis, but it bears great promise in multiple applications of cancer therapies. Especially, due to the high stability of miRNAs and advance of RNA purification techniques, miRNAs can be extracted from not only tumors samples exposed to variable treatments, including formalin-fixing and paraffin-embedding [40], but also serum and urine [41–44].

### 2.2. MicroRNAs in Oncogenesis

Many miRNAs have been shown to regulate cell survival and proliferation, angiogenesis, and epithelial-mesenchymal transition (EMT). The miRNAs associated with oncogenesis are also known as “oncomirs”. Depending on their major targets, oncomirs can be classified into the oncogenic and tumor suppressive miRNA groups. As the classification implies, tumor suppressive miRNAs repress protein-coding oncogenes whereas oncogenic miRNAs repress protein-coding tumor suppressors. Some miRNAs display both oncogenic and tumor suppressive activities, depending on the tissue and tumor contexts. Wang *et al.* demonstrated that oncogenic and tumor suppressor miRNAs show clearly different patterns in many aspects, including evolutionary rates, expression patterns, chromosome distribution, molecule sizes and of course targets [33].

An example of miRNAs that have opposing effects is the miRNA-17-92 cluster that has been implicated in regulating cellular survival. The polycistronic miRNA-17-92 cluster (miR-17-92) is located on chromosome 13 open reading frame 25 (c13orf25) in the human genome and contains seven miRNAs (miR-92-1, miR-19a, miR-20a, miR-19b, miR-18a, miR-17-5p and miR-17-3p) [45]. Ota *et al.* reported that the chromosomal region 13q31-q32, where the miR-17-92 cluster resides, is amplified in malignant lymphoma [46]. He *et al.* observed that five miRNAs (miR-92-1, miR-19a, miR-20a, miR-19b and miR-17-5p) from this cluster are up-regulated in human B-cell lymphoma and cell lines [47]. Ectopic expression of this miRNA cluster promotes c-Myc-induced lymphomagenesis in mice suggesting the cooperation between mir-17-19b and c-Myc in oncogenesis. In another study, O’Donnell *et al.* identified an additional miRNA, miR-18a, at the miRNA-17-92 locus and demonstrated that c-Myc transcriptionally activates the expression of these 6 miRNAs (miR-92-1, miR-19a, miR-20a, miR-19b, miR-17-5p and miR-18a) [48]. Interestingly, miR17-5p and miR-20a negatively regulate E2F1 expression, which is also activated by c-Myc. E2F1 is a crucial driver of cell cycle progression from the G1 to S phase, but its expression can induce either pro-metastatic activity or cell apoptosis depending on its molecular contexts [49,50]. Thus, the ectopic miR-17-92 expression in a c-Myc background confers an overall pro-survival trait to the cells through eliminating E2F1-mediated apoptotic potential. This is corroborated by the aforementioned studies of He *et al.*, in which c-Myc and miR-19b overexpression promoted murine lymphomas without causing any indicative sign of apoptosis [47]. The presence of this interactive regulatory network suggests a tight control of c-Myc-mediated proliferation signal during oncogenesis.

Data from several other groups also suggest tumor suppressive activities of the miRNAs in this cluster. Hossain *et al.* reported that miR-17-5p acts as a tumor suppressor through repressing the expression of AIB1 (or NCOA3) and consequently reducing the proliferation of breast cancer cells [51]. AIB1 was named for its “amplified in breast cancer” and has been suggested to play an oncogenic role in mammary oncogenesis [52,53]. Interestingly, AIB1 functions as a coactivator of E2F1 to promote breast cancer cell proliferation [52]. Thus, through repressing both AIB1 and E2F1, miR-17-5p may “kill two birds with one stone” in reducing breast cancer cell proliferation.

Yu *et al.* reported a negative feedback regulation between miR-17-5p/miR-20a and cyclin D1 [54], an oncoprotein in breast cancer. Cyclin D1 inversely correlated with miR-17-5p/miR-20a levels in breast cancer samples and cell lines. In a negative regulatory loop, cyclin D1 induces miR-17-5p/miR-20a expression, while these two miRNAs target the cyclin D1 3′-UTR to limit its proliferative activities. The same group also reported that miR-17-5p/miR-20a repress cytokeratin 8 through inhibiting cyclin D1 [55]. Additionally, these two miRNAs target the 3′-UTR of the interleukin-8 mRNA. Thus, miR-17-5p/miR-20a regulates both cellular secretion and tumor microenvironment to block migration and invasion of neighboring cells in breast cancer [55]. The seventh member of this cluster, miR-17-3p, is the “passenger strand” of miR-17-5p that is also processed into a mature miRNA. Recent studies indicate that it represses the expression of vimentin and Mdm2, suggesting a tumor suppressive role in oncogenesis [56,57].

Recent studies revealed a tumor suppressive role of miR-101 in a variety of cancers, including prostate, bladder, breast and gastric cancers [58–62]. MiR-101 is frequently downregulated in these cancers compared to their normal adjacent tissues. On the other hand, ectopic expression of miR-101 in various cancer cell lines resulted in decreased cellular proliferation, motility and invasiveness, indicating its antitumoral activities. The tumor suppressive function of miR-101 has been corroborated by its inhibitory effect on EZH2, an oncogene of many solid tumors [63]. Varambally *et al.* reported the miR-101 loss resulted in increased EZH2 expression [62]. Importantly, genomic loss of the miR-101 locus correlates with EZH2 overexpression in solid tumors. Cao *et al.* further demonstrated that miR-101 negatively regulates EZH2 expression in prostate cancer cells, while miR-101 expression is modulated by androgen receptor and HIF-1α/HIF-1β [58]. The negative regulation of EZH2 by miR-101 has been confirmed by the studies from several other groups [64–66]. Consistent with these observations, a recent report suggested a positive regulation of E-cadherin by miR-101 [67]. E-cadherin is frequently downregulated in aggressive cancers and its loss increases cell dissemination and cancer cell invasiveness. In the study by Qazi *et al.*, ectopic miR-101 restored E-cadherin expression in pancreatic ductal adenocarcinoma cells by reducing EZH2-mediated histone H3-K27 methylation. Overall, these studies suggest that miR-101 primarily targets oncogene EZH2 and its deletion contributes to oncogenesis through aberrant epigenetics caused by EZH2 overexpression.

The let-7 family was one of the first mammalian miRNAs to be discovered and consists of 13 members located on different genomic loci that are often lost in human cancers [68]. Reduced expression of the let-7 members is associated with more dedifferentiated and aggressive cancers [69]. These observations suggested a possible tumor suppressive role of the let-7 family members. Recent studies indicated that let-7 can target oncogenes RAS and c-Myc that have multiple potential binding sites of let-7 miRNAs in their 3′-UTRs. Johnson *et al.* detected reduced let-7 expression in tumor tissues compared to normal adjacent tissues in lung cancer samples [70]. Interestingly, let-7 miRNA levels inversely correlated with RAS protein expression but not its mRNA levels, suggesting a mechanism of let-7-mediated translational inhibition without mRNA degradation. Overall, let-7 miRNAs may target several key oncogenes, such as RAS and c-Myc, to suppress the proliferative signals from these two oncogenes.

Many other miRNAs have been demonstrated to play a role in cancer development and progression. It is noteworthy that reports from different groups may present paradoxical results. These discrepancies could result from different experimental settings and also reflect the complexity of miRNA-regulated network in cancers. As discussed above, each miRNA potentially target hundreds genes and its ectopic overexpression may perturb multiple cellular processes leading to artificial phenotypic changes. Thus, studies only using miRNA overexpression or reporter assay without any miRNA depletion or tumor sample correlation studies may not truly represent physiological relevance. Nevertheless, currently available literature unequivocally indicates the prognostic potential and biological activities of miRNAs in different cancers.

### 2.3. P53 Is a Key Regulator of MicroRNAs Biogenesis

**Figure 2:**
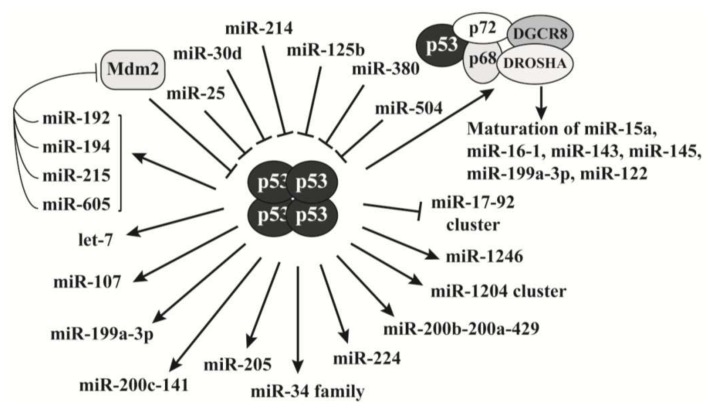
The interplay between miRNAs and p53. While p53 regulates the gene expression of many miRNAs, its expression is also inhibited by miRNAs. When cells are exposed to genotoxic stresses, p53 activates four miRNAs that repress Mdm2 expression, which leads to p53 accumulation and cell cycle arrest or apoptosis. P53 protein also associates with the DROSHA complex to directly regulate miRNA maturation.

The tumor suppressor p53 is one of the best-studied proteins in the field of cancer research. Owing its well-characterized regulation, the role of p53 in the miRNA network has been extensively explored in the past decade. As a transcription factor, p53 forms tetramers to bind to its consensus sites on target genes and mediates their transcription. Increasing evidence shows that p53 exerts its antiproliferative activities at least partially through the transcriptional regulation of miRNA expression (Figure 2), in addition to its canonical tumor suppressive role [20,71]. The best characterized p53 target is the miR-34 family [72]. The miR-34 family includes miR-34a, -34b and -34c. MiR-34a is transcribed by chromosome 1, and miR-34b and -34c are transcribed by two proximal loci on chromosome 11 and controlled by the same promoter. These miRNAs exert tumor suppressive activities through repressing proliferative genes, such as c-Myc and BCL2, and their overall function is inducing apoptosis, cell cycle arrest or senescence [73–75]. Consistently, they are frequently silenced by promoter methylation in tumors [76]. Thus, the p53-promoted expression of the miR-34 family extended its activated tumor suppressive network.

P53 also stimulates the expression of many other miRNAs that have antiproliferative activities. For example, p53-mediated miRNA expression plays a role in hypoxia. During hypoxia of tumor cells, the upregulated hypoxia inducing factor (HIF)-1α forms a heterodimer with HIF-1β to become a transcription factor HIF-1 that activates pro-angiogenic genes such as vascular endothelial growth factor A (VEGFA), to promote angiogenesis for tumor growth and metastasis [77,78]. Yamakuchi *et al.* reported that miR-107 reduces hypoxia signaling by inhibiting HIF-1α expression in human colon cancer cells [79]. As a transactivator, p53 promotes the expression of miR-107 to reduce HIF-1α levels, which quenches the hypoxic signal to block tumor angiogenesis. Among other p53-activated miRNAs, miR-145 represses c-Myc expression [80], and miR-200c/miR-141 and miR-200b/miR-200a/miR-429 inhibit EMT through downregulating ZEB1 and ZEB2 [81,82]. As discussed above, miR-17-92 cluster exhibits proliferative activities based on most literature. P53 represses the expression of the miR-17-92 cluster, opposite to its transactivating effects on most other miRNAs (Figure 2), and this regulation likely contributes to the p53-induced apoptosis [83].

In addition to transcriptional regulation, p53 is directly involved in the maturation process of miRNAs. DROSHA is a major component of the complex that processes pri-miRNA into pre-miRNA. P53 associates with DROSHA in a RNA dependent manner and facilitates DROSHA-mediated pri-miRNA processing [84]. Interestingly, although this process is independent of its transcriptional activity, transcriptionally inactive p53 mutants do not show this capability. Actually, these p53 mutants interfere with the assembly of the DROSHA complex and consequently attenuate miRNA processing. Currently, p53 has been reported to regulate the maturation of at least 6 miRNAs (miR-15a, miR-16-1, miR-143, miR-145, miR-199a, and miR-122) [84–87]. Whether p53 is involved in the processing of other miRNAs remains to be determined.

Conversely, p53 expression can also be regulated by miRNAs (Figure 2). The functions of these miRNAs are not uniformly oncogenic; thus, whether their role in repressing p53 expression is physiologically significant is unclear. There is a negative feedback loop between p53 and the ubiquitin E3 ligase Mdm2. While Mdm2 stimulates p53 ubiquitination and degradation, p53 activates Mdm2 gene expression. This feedback regulation is important to maintain p53 homeostasis in normal cells, which is disrupted when exposed to genotoxic stresses [88]. Recent studies reveal that some miRNAs can break this negative feedback loop leading to p53 accumulation. Pichiorri *et al.* reported that p53 activates the expression of miR-192, miR-194 and miR-215 and these miRNAs target the Mdm2 3′-UTR to repress its expression; thus, downregulation of p53-inducible miR-192, miR-194 and miR-215 causes aberrant Mdm2 increase and p53 downregulation in multiple myeloma [89]. Consistently, miR-192, miR-194 and miR-215 are downregulated in multiple myeloma and renal cancers [89–91]. Xiao *et al.* demonstrated that p53 activates miR-605 that also represses Mdm2 expression [92]. Thus, p53 activates these four miRNAs to downregulate its negative regulator Mdm2 leading to p53 accumulation in response to stress.

### 2.4. C-Myc Regulates the Synthesis of miRNAs

The oncogene c-Myc is a transcription factor and mediates target gene expression through recruiting various chromatin modifiers [93]. Recent studies reveal its role in regulating miRNA synthesis. Interestingly, the effects of c-Myc-mediated transcription of many miRNAs are opposite to those regulated by p53.

**Figure 3:**
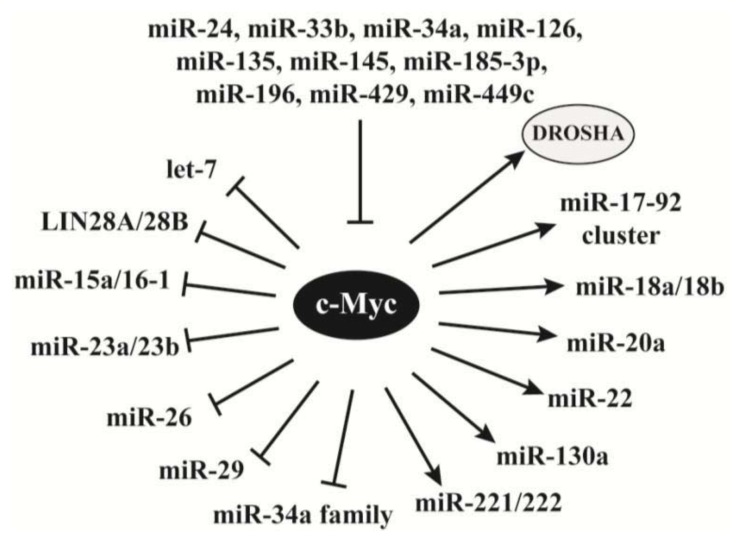
The interplay between miRNAs and c-Myc. While c-Myc regulates the expression of multiple miRNAs, its expression is inhibited by different miRNAs. C-Myc also activates the gene expression of DROSHA to directly promote miRNA procession.

C-Myc represses the expression of multiple miRNAs (Figure 3). As discussed above, p53 transactivates the miR-34 family that represses c-Myc expression [72,75]. On the other hand, c-Myc represses the expression of the miRNAs in the miR-34a family [94]. C-Myc also recruits HDAC3 to downregulate the expression of miR-15a/miR-16-1 [95] that block the expression of multiple oncogenes, such as BCL2 and cyclin D1 [27,96]. Similarly, c-Myc also recruits HDAC3 and EZH2 to silence the expression of miR-29 [97] that has been defined as a tumor suppressor [98]. Among other c-Myc repressed miRNAs, let-7 inhibits androgen receptor and KRAS [68,99], miR-23a/miR-23b block glutaminase [100], and miR-26 targets EZH2 and cyclin D2 [58]. Thus, c-Myc-mediated repression of these miRNAs can release the expression of multiple oncogenes or proliferative genes to promote oncogenesis.

c-Myc also activates the expression of multiple miRNAs. Similar to its role in antagonizing p53-mediated miR-34 miRNA expression, c-Myc promotes the transcription of miR-17-92 cluster [101] that is downregulated by p53. Kim *et al.* carried out a combined analysis of mRNA and miRNA expression profiles that revealed multiple c-Myc-induced miRNAs and their downstream targets [102]. Among their discovered miRNAs, miR-221 and miR-222 have been shown to target p27, p57 and PTEN, and exhibit proliferative activities [103–106]. For the other c-Myc-activated miRNAs, the authors predicted that miR-20a targets p21, RB1, PTEN and interferon regulatory factor 1 (IRF-1), and miR-130a inhibits tuberous sclerosis 1 (TSC1) and CYLD [102]. While p53 protein direct associates with the miRNA processing machinery, c-Myc activates DROSHA expression through binding to the E-box in its promoter and consequently facilitates miRNA processing [107].

While c-Myc acts as a regulator of miRNAs, its expression has been reported to be modulated by a number of miRNAs (Figure 3). Among them, miR-34a, miR-126 and miR-145 have been documented as tumor suppressors [33].

## 3. Long Noncoding RNA

Long noncoding RNAs (lncRNAs) were previously defined as RNA molecules longer than 200 nucleotides that are not translated into proteins [6,108]. Recently, Spizzo *et al.* amended this definition linking to the biological functions and described that lncRNAs are a class of RNA molecules that do not fit into any known class of small and structural RNAs, and possess regulatory roles in their primary or spliced form [109]. The GENCODE Consortium is a part of the ENCODE (ENCyclopedia Of DNA Elements) project and aims to identify all gene features in the human genome. The GENCODE 7 release in 2012 indicates that the human genome contains at least 9,640 long noncoding RNA loci that can potentially encode 15,512 transcripts [110]. LncRNA production is regulated by mechanisms similar to these of protein-coding genes, such as histone modifications and RNA splicing, and their expression shows tissue-specific patterns [111]. Most lncRNAs are localized in nucleus and associated with chromatin, and some of them are preferentially processed into small RNAs [111]. Banfai *et al.* demonstrated that lncRNAs are rarely translated in two tested human cell lines, suggesting that ribosomes can differentiate the coding and noncoding transcripts for translation [112].

Recent studies continue elucidating novel and unpredicted biological activities of the lncRNAs, which were previously ascribed as protein functions. Based on the regulatory mechanisms, the lncRNAs can be divided into four categories, including the lncRNAs that (1) regulate gene expression, (2) act as miRNA decoys to free target mRNAs, (3) regulate mRNA translation, and (4) regulate protein activities.

### 3.1. LncRNAs Regulating Gene Expression

The X-inactive-specific transcript (Xist) was one of the first lncRNAs discovered in mammals [113]. Xist is encoded by the inactive X chromosome (Xi) and the genomic locus can transcribe a 17- to 20-kb RNA. This lncRNA binds the Xi *in cis* to induce chromosome X silencing by recruiting the polycomb repressive complex 2 (PRC2) that induces histone H3-K27 methylation, a hallmark of gene inactivation [114,115]. In this process, the transcription repressor Yin Yang 1 (YY1) confers allele-specific binding of Xist to the Xi with the involvement of two other noncoding RNAs, Jpx [116] and Ftx [117]. There is another lncRNA, Tsix, that is transcribed at the same locus of Xist but in the reverse direction and thus antisense to Xist [118]. Tsix regulates imprinted and random X inactivation in development [119]. An additional Xist-related RNA transcript is Xite that also plays a role in the X chromosome inactivation [120].

The association of epigenetic silencing complexes with Xist to induce transcriptional silencing has been extended to several recently characterized lncRNAs, which also associate with the PRC2 and other chromatin repressive complexes [121,122]. One of these lncRNAs is HOX Antisense Intergenic RNA (HOTAIR) that is a 2.2-kb transcript located at the HOXC gene cluster on chromosome 12 [122]. HOTAIR also modulates gene expression through epigenetic regulation. Unlike Xist that acts *in cis*, HOTAIR functions *in trans* to recruit the PRC2 to the *HOXD* locus on chromosome 2 to induce transcriptional silencing [122]. In addition to associating with PRC2, HOTAIR also interacts with the LSD1/CoREST/REST histone modification complex, leading to both histone H3-K27 methylation and H3-K4 demethylation [123]. Since HOTAIR plays an important role in the epigenetic regulation of its target genes, it is not surprising that its deregulation has been observed in different types of cancers. Recent studies suggest that HOTAIR overexpression is positively associated with increased tumor cell malignancy. Gupta *et al.* reported that HOTAIR is overexpressed in both primary and metastatic breast cancer tissues, and its levels in the primary tumors could be used as a significant predictor of subsequent tumor metastasis and survival of the patients [124]. Ectopically expressed HOTAIR could confer the breast epithelial cancer cells with invasive and metastatic potential while its depletion in breast cancer cells abrogated these activities [124]. The role of HOTAIR in promoting oncogenesis has also been reported in other cancers. Yang *et al.* compared HOTAIR levels between tumorigenic and adjacent non-tumorigenic tissues of hepatocellular carcinoma (HCC) samples and found that this lncRNA was expressed at higher levels in malignant tissues [125]. In addition, the survival analysis of a cohort consisting of 60 HCC patients revealed that high HOTAIR expression could serve as an independent prognostic marker for disease recurrence and reduced patient survival [125]. Another HCC-related study suggested that HOTAIR expression is a potential biomarker for lymph node metastasis from the primary tumors [126]. Furthermore, HOTAIR upregulation was also observed in a cohort of patients diagnosed with the stage IV colorectal cancer (CRC) [127]. In this study, Kogo *et al.* indicated that high HOTAIR expression showed significantly positive correlation with the liver metastasis and poor patient outcome, further supporting that HOTAIR expression is a potential prognostic marker of multiple cancers. In pancreatic cancer, HOTAIR levels were also increased in tumorigenic tissues compared to the non-tumorigenic tissues, and associated with a more aggressive phenotype [128]. The oncogenic role of HOTAIR in pancreatic cancer cell invasion was validated by its siRNA-mediated knockdown and overexpression studies [128], consistent with the observations in the other aforementioned cancers. Interestingly, gene array studies showed only a small overlap of HOTAIR-regulated genes between pancreatic cancer and breast cancer [128], suggesting that HOTAIR may regulate different sets of target genes in a cell type-specific manner.

EZH2 is a core component of the PRC2. EZH2 knockdown followed by chromatin immunoprecipitation demonstrated that HOTAIR-mediated gene repression could be either PRC2-depedent or -independent in pancreatic cancer cells, although the PRC2 is necessary for HOTAIR target gene repression in breast cancer cell [124,128]. This discrepancy between the two cancer types suggests the presence of yet unidentified epigenetic mechanisms regulating HOTAIR-mediated transcriptional silencing.

Wang *et al.* identified the lincRNA HOTTIP that is transcribed from the 5′ tip of the HOXA locus and modulates the activity of the WDR5-MLL complex, in which the WD40-repeat protein WDR5 binds the MLL complex to activate its histone H3-K4 methyltransferase activity [129]. Chromosomal looping can make HOTTIP stay in the vicinity of its target genes and let it bind WDR5 to promote the WDR5/MLL complexes-mediated histone H3-K4 methylation, which leads to target gene activation. Thus, HOTTIP serves as a key intermediate to transmit information from higher order chromosomal looping into chromatin modifications [129]. Another HOXA-related lncRNA is HOX antisense intergenic RNA myeloid 1 (HOTAIRM1) that is transcribed at a direction antisense to the HOXA gene [130]. The knockdown of HOTAIRM1 reduced the expression of HOXA1 and HOXA4 during the myeloid differentiation in promyelocytic leukemia cells. Whether this lncRNA acts as a miRNA decoy to promote the expression of these HOX genes remains to be determined.

Metastasis-associated lung adenocarcinoma transcript 1 (MALAT1), also known as nuclear-enriched abundant transcript 2 (NEAT2), is one of the first identified cancer-associated lncRNAs [131]. MALAT1 is a highly conserved noncoding transcript of over 8000 nts encoded by a locus on chromosome 11. It was initially recognized as a prognostic marker of increased metastatic risk for the patients of non-small cell lung carcinoma (NSCLC). Subsequent studies revealed that MALAT1 is localized in nuclear structures enriched with splicing and transcription factors, known as nuclear speckles, suggesting that this lncRNA may modulate alternative splicing of target genes [132,133]. Using RNAi-mediated depletion for the components of the nuclear speckles, Miyagawa *et al.* demonstrated that the pre-mRNA splicing activator RNPS1, the splicing coactivator SRm160 and the spliceosomal intron binding protein IBP160 promote MALAT1 localization to the nuclear speckles [134]. Furthermore, MALAT1 depletion and delocalization from the nuclear speckles resulted in downregulation of two interferon-induced genes (OASL and IFI44) and a potential celiac disease susceptibility gene, SPINK4 [134].

Despite these studies suggesting a role of MALAT1 in RNA splicing, a recent study by Gutschner *et al.* showed that this lncRNA regulates gene expression but not alternative splicing in lung cancer cells [135]. MALAT1 knockout was achieved by genomic integration of RNA destabilizing elements using zinc finger nucleases, leading to 1000-fold MALAT1 reduction. Using these MALAT1-knockout cells, the authors demonstrated that MALAT1 represses anti-metastatic genes and activates pro-metastatic genes in lung cancer cells, but does not affect genes regulating cell growth. In a xenograft mouse model, the MALAT1-depleted lung cancer cells showed reduced tumor formation compared to the cells with the intact MALAT1. This observation is in contrast to the findings reported by Yang *et al.* showing that MALAT1 cooperates with Polycomb 2 protein (Pc2) in regulating the activation of the growth-control gene program in 293T cells [136]. The different cell types employed in their experiments might contribute to the discrepancy between the two studies. Consistent with this prediction, MALAT1 is widely expressed in most normal human tissues, such as pancreas and lung, but absent in several other tissues including skin, stomach, bone marrow and uterus [131]. This suggests that this lncRNA may possess tissue specific functions. In addition to its role in regulating lung cancer metastasis, MALAT1 is upregulated in uterine endometrial stromal sarcoma [137], cervical cancer [4] and hepatocellular carcinoma [138], while its expression in the corresponding healthy tissues is undetectable or intermediate [131]. Although the oncogenic role of MALAT1 in different cancers has been demonstrated by correlational and functional studies, the molecular mechanisms underlying its activities in regulating gene expression and RNA splicing remain undetermined. To date, several studies suggested the essential role of the 3′ end sequence and structure to its metastasis-promoting function, nuclear localization and stability [139–142].

ANRIL is a large antisense ncRNA of the INK4b/ARF/INK4a locus [143]. Yap *et al.* demonstrated that ANRIL binds chromobox 7 (CBX7), a component of the polycomb repressive complex 1. This interaction contributes to the role of CBX in promoting EZH2-mediated H3-K27 methylation at the INK4b/ARF/INK4a locus and consequently represses the tumor suppresser INK4a gene. Consistently, both CBX7 and ANRIL are increasingly expressed in prostate cancer [144].

GAS5 (growth arrest-specific transcript 5) is a lncRNA regulating growth arrest of T-cells and lymphocytes [145]. Ectopic GAS5 increases apoptosis and reduces cell cycle progression. Consistently, its downregulation inhibits apoptosis and promotes cell cycle. Kino *et al.* investigated the mechanism underlying the growth suppressive activities of GAS5 and discovered its role in blocking gene expression mediated by glucocorticoid receptor (GR) [146]. GAS5 binds to the DNA-binding domain of GR and thus prevents its association with the glucocorticoid response element of the GR target genes with anti-apoptotic activities, such as inhibitor of apoptosis 2 (cIAP2). Thus, abundantly expressed GAS5 during starvation can sensitize the cells to apoptosis. The nonsense-mediated mRNA decay (NMD) is a system that controls the quality of gene transcripts and reduces errors in gene expression by eliminating RNAs with premature stop codons [147]. Meanwhile, this mechanism also regulates the abundance of cellular transcripts, including ncRNAs. Recently, Zhang *et al.* demonstrated a reciprocally negative regulation between GAS5 and miR-21 [148]. While miR-21 represses GAS5 by targeting a sequence encoded by its exon 4, GAS5 inhibits miR-21 expression. Thus, GAS5 antagonizes the oncogenic activity of miR-21 [149] through reducing its cellular levels. Consistently, miR-21 and GAS5 showed negative correlation in breast cancer specimens [148]. The tumor suppressive role of GAS5 is supported by the identification of genetic susceptibility of its genomic locus, 1q25, to several cancers, including melanoma [150], prostate cancer [151], breast cancer [152,153], colorectal cancer [154] and B-cell lymphoma [155]. Additionally, Tani *et al.* indicated that GAS5 can be stabilized with the depletion of UPF1, an essential component of NMD or during starvation [156] and the GAS5 introns encode multiple snoRNAs [157–160].

Several other lncRNAs have also been demonstrated to regulate chromatin remodeling and gene transcription. PTENP1 is the PTEN pseudogene and encodes two antisense RNA (asRNA) transcripts, asRNA α and β [161]. The α asRNA isoform can recruit DNMT3A, EZH2 and G9A to the PTEN promoter and repress its transcription. This will be further discussed below with other regulatory mechanisms of PTENP1. Evf2 is a polyadenylated lncRNA identified in embryonic brain cells [162]. This lncRNA regulates the transcription of homeodomain transcription factors DLX5 and DLX6 through recruiting DLX and MECP2 to the DNA regulatory elements in the intergenic region of these two genes. As a p53 transactivated lncRNA, lincRNA-p21 is a key mediator of p53-dependent gene repression through a mechanism of recruiting heterogeneous nuclear ribonucleoprotein K (HNRNP) to these p53 target genes [163]. Thus, inhibition of lincRNA-p21 affects the expression of a number of p53 repressed genes. Sheik *et al.* identified an Oct4-activated lncRNA, AK028326, and discovered that this lncRNA regulates pluripotency in mouse embryonic stem cells [164]. Interestingly, AK028326 activates Oct4 expression in a regulatory feedback loop.

### 3.2. LncRNAs Acting as miRNA Decoys to Free Target mRNAs

Since the regulation of gene expression by miRNAs was revealed, researchers have been using miRNA sponges, RNA molecules containing the target sequence or reverse complementary sequence of a miRNA to be sponged, as a tool to inhibit the function of miRNAs and release their target gene expression [165]. Recent studies suggest that this approach naturally exists in cancers to modulate tumor suppressor and oncogene levels. In some literature, these decoy RNAs are also named as competitive endogenous RNAs (ceRNAs).

**Figure 4:**
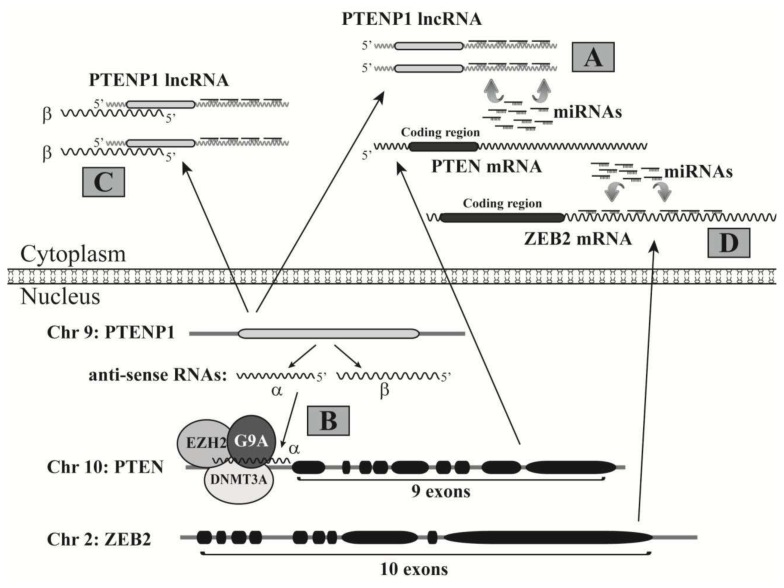
PTEN expression is regulated by multiple ncRNAs. (**A**) The PTEN pseudogene, PTENP1, can transcribe into the PTENP1 lncRNA that acts as a decoy to sponge the miRNAs targeting at the 3′-UTR of the PTEN mRNA; (**B**,**C**) The locus of the PTEN pseudogene can also transcribe from the reverse direction to make two antisense RNA (asRNA) isoforms, α and β; (**B**) The α asRNA isoform binds the PTEN promoter and recruits DNMT3A, EZH2 and G9A, which causes epigenetic silencing of the PTEN gene; (**C**) The β asRNA isoform associates with the PTENP1 lncRNA to increase its stability and miRNA decoy activity; (**D**) The ZEB2 mRNA has a long 3′-UTR with many binding sites of miRNAs that also potentially target the PTEN 3′-UTR. Thus, the ZEB2 mRNA acts as a decoy to sponge many miRNAs and consequently promotes PTEN expression.

PTEN (phosphatase and tensin homolog) is a well characterized tumor suppressor with phosphatase activity. It is encoded at the 10q23.3 locus on chromosome 10 and frequently inactivated through diverse mechanisms in human cancers, highlighting its crucial role in oncogenesis. The 3′-UTR of the PTEN mRNA has 3,329 nts, markedly longer than the average 3′-UTR length (740 nts) of eukaryotic mRNAs [166], implicating its vulnerability as a target of miRNAs. Thus, while PTEN inactivation can be achieved by gene deletion and epigenetic silencing in cancers, its expression is also regulated by multiple miRNAs. For example, the PTEN 3′-UTR contains potential binding sites for over 10 miRNAs overexpressed in glioblastoma multiforme, which is more than 2 times higher than any other tumor suppressor [167]. PTEN expression can be repressed by miR-21, miR-221 and miR-222 [106,168]. Recent studies revealed novel mechanisms regulating PTEN expression through its pseudogene PTENP1 (also called PTH2 or ψPTEN). Pseudogenes are dysfunctional relatives of their cognate genes but have lost the protein-coding ability due to premature stop codons, deletions/insertions or frameshift mutations, and thus cannot be translated into functional proteins [169]. The PTEN pseudogene PTENP1 is highly transcribed in certain tissues and cells, suggesting that this lncRNA may have biological activities [170]. The functional relationship between the PTEN and its pseudogene was first discovered by Poliseno *et al.* [171]. In their study, the authors demonstrated that PTENP1 modulates endogenous PTEN transcript levels by acting as a molecular sponge for PTEN-targeting miRNAs; thus, the PTENP1 transcript serves as an effective decoy and exerts tumor suppressive functions (Figure 4). This novel regulatory role of the PTENP1 can be extended to KRAS1P, the pseudogene of the oncogene KRAS. In a study by Poliseno *et al.*, the overexpression of KRAS1P led to increased KRAS mRNA levels in prostate cancer DU145 cells through a mechanism of sequestering KRAS-targeting microRNAs, and consequently promoted cell proliferation [171].

As briefly discussed above, a recent study by Johnsson *et al.* provided evidence of another regulatory mechanism of PTEN by its pseudogene PTENP1 (Figure 4). The authors discovered that the PTENP1 locus can be transcribed from a reverse direction to create two isoforms of an antisense RNA (asRNA), α and β [161]. The α isoform of the asRNA binds the PTEN promoter and recruits DNMT3A and two histone methyltransferases EZH2 and G9A, which mediate the methylation of histone H3-K27 and H3-K9, respectively, two well-characterized markers of gene repression [172]; thus, the PTENP1 α asRNA negatively regulates PTEN gene expression through promoting the epigenetic silencing of its promoter. The β asRNA isoform interacts with the PTENP1 lncRNA through RNA-RNA pairing, which can maintain stable PTENP1 lncRNA levels in cytoplasm, increase its stability and facilitate its role as a microRNA sponge; thus, the PTENP1 β asRNA activates PTEN expression through facilitating the decoy activity of the PTENP1 lncRNA. Overall, the PTENP1 α and β asRNAs exhibit oncogenic and tumor suppressive roles, respectively, based on their effects on PTEN expression (Figure 4). Whether the two asRNA isoforms are differentially expressed in human cancers remains to be determined.

Interestingly, PTEN expression can also be regulated at the translational level by the sponge effect of the transcript from another gene. The full length ZEB2 mRNA has a very long 3′-UTR (over 5,000 nts) and contains multiple potential binding sites of miRNAs that can also target the PTEN 3′-UTR, such as miR-181a and miR-200/miR-141 [173]. Thus, the ZEB2 mRNA serves as a decoy or ceRNA of the PTEN mRNA (Figure 4) and reduced ZEB2 expression activates the PI3K/AKT pathway through downregulating PTEN.

There are several other examples showing that lncRNAs act as decoys to stabilize mRNAs. BACE-AS regulates the expression of β-secretase-1 (BACE1), a crucial enzyme in Alzheimer’s disease pathophysiology [174]. Linc-MD1 is a muscle-specific lncRNA and activates the expression of MAML1 and MEF2C through its decoy role for miR-133 [175].

### 3.3. LncRNAs Regulating mRNA Translation

As discussed above, MALAT1 was initially demonstrated to possess activities in regulating alternative splicing. MALAT1 associates with serine/arginine (SR) splicing factors at the nuclear speckle domains of nucleus and is involved in the process of alternative splicing [133]. Thus, MALAT1 depletion or ectopic SR protein expression affects the alternative splicing of a similar set of pre-mRNAs. Additionally, MALAT1 alters the phosphorylation of the SR splicing factors, which is essential to their activities in regulating alternative splicing. However, this regulation may only be present in specific cell types or under particular conditions, because MALAT1-knockout mice were viable and fertile, and showed regularly localized nuclear speckle markers [176]. Whether this regulatory mechanism contributes to the activity of MALAT1 in promoting tumor metastasis has not been determined.

A recent study from Zhang *et al.* demonstrated the lncRNA, lincRNA-RoR, acts as a strong negative regulator of p53 [177]. LincRNA-RoR reduces p53 expression in cells exposed to DNA damage stress, but not in unstressed cells, through directly binding to the heterogeneous nuclear ribonucleoprotein I (hnRNPI) and repressing p53 mRNA translation. As an autoregulatory feedback regulation, p53 transcriptionally induces lincRNA-RoR expression. The functional interplay between lincRNA-RoR and p53 may serve as additional surveillance to mediate cell response to genotoxic stresses.

As discussed above, another p53-transactivated lncRNA, lincRNA-p21, is involved in p53-dependent gene transrepression [163]. A recent study from Yoon *et al.* demonstrated that lincRNA-p21 also modulates translation [178]. This lncRNA can associate with JunB and β-catenin mRNAs to reduce their translation rates. A RNA-binding protein, HuR, can recruit let-7/AGO2 to lincRNA-p21 to reduce its stability; thus, elevated HuR releases lincRNA-p21-mediated repression of JunB and β-catenin expression. Since lincRNA-p21 is transactivated by p53, the negative regulation of JunB and β-catenin by lincRNA-p21 is consistent to the tumor suppressive role of p53.

### 3.4. LncRNAs Regulating Protein Activities

Telomeres are the DNA-protein complexes at the end of eukaryotic chromosomes and essential to chromosome stability. As a frequently activated reverse transcriptase, telomerase adds DNA sequence repeats (“TTAGGG” in vertebrates) to the telomere regions of chromosome to maintain the telomere length. Azzalin *et al.* discovered that telomeres can be transcribed into telomeric repeat-containing RNA (TERRA) [179]. These molecules have different lengths and were predicted to play a role in the maintenance of telomere integrity through an unclear mechanism. A later study from Redon *et al.* provided a possible mechanism underlying this activity of TERRA [180]. The authors demonstrated that TERRA binds telomerase and thus acts as a potent competitive inhibitor for the telomeric DNA. Consistently, TERRA expression is significantly downregulated in multiple tumor cell lines, which plays a role in telomere maintenance and cell immortalization of cancer cells.

Wang *et al.* demonstrated an indirect regulation of the histone acetyltransferase activities of CBP and p300 by ncRNAs [181]. The 5′-regulatory region of the cyclin D1 gene encodes at least four ncRNAs. In response to ionizing radiation, these ncRNAs bind TLS (translocated in liposarcoma) protein at the chromatin of the cyclin D1 promoter region and cause an allosteric effect on this protein. This TLS conformational change promotes its activity of inhibiting CBP/p300-mediated histone acetylation and consequently silences cyclin D1 gene. These data indicate that ncRNAs encoded by a promoter can act as selective ligands to modulate the activities of transcription cofactors in response to genotoxic stresses.

LncRNAs can also regulate protein activity through altering their subcellular localization. Nuclear factor of activated T-cells (NFAT) represents a family of transcription factors regulating immune response. Some of NFAT proteins, such as NFAT1 and NFAT5, contribute to tumor metastasis and cell motility [182,183]. The noncoding repressor of NFAT (NRON) is a ncRNA associated with multiple proteins [184]. NRON binds phosphorylated NFAT1 to sequester NFAT in the cytoplasm. The depletion of NRON leads to NFAT dephosphorylation and nuclear import. Thus, ncRNAs can be a part of a scaffold to trap a latent transcription factor [185] and regulate the expression of its target genes.

## 4. Circular RNAs

Recent advances in high-throughput sequencing coupled with powerful computational analyses of expression data have allowed researchers to identify and characterize new RNA species, one of which is a new class of RNA molecules, circular RNAs (circRNAs), present in humans and animals. CircRNAs were first discovered in plants and considered as viroids due to their predicted role as subviral agents [186]. These covalently linked and single-stranded ncRNAs have molecular weights of over 100,000 Da and are highly thermal stable. Initially, most circRNA molecules were dismissed as transcription noise or by-products generated by cellular splicing machineries. However, this notion has been challenged by emerging studies suggesting that some circRNAs are evolutionary conserved in human and mice [14,187]. The exact mechanism of circRNA biogenesis remains to be elucidated. Salzman *et al.* indicated that circRNAs are produced by a non-canonical mode of RNA splicing [188]. Several other studies suggested that circRNA may be at least partially contributed by exon-skipping events, which can create an exon-containing lariat and then possibly undergo internal splicing to generate an exon circle [187,189,190].

In 2011, Hansen *et al.* reported the miRNA decoy function of a noncoding circRNA [191]. In this study, the authors demonstrated that miR-671 directly targets and cleaves a circular antisense transcript of the cerebellar degeneration-related protein 1 (CDR1) in an AGO2-dependent manner. In 2013, thousands of well-expressed and stable circRNAs with tissue- and developmental stage-specific expression were identified [14]. The antisense to the CDR1 transcript (CDR1as) is densely bound by Argonaute proteins and contains 63 conserved binding sites of miR-7, suggesting that this circRNA functions as a miRNA sponge of miR-7 to release its target gene expression [14,190]. Indeed, using a zebrafish model, Memczak *et al.* demonstrated that introduction of human CDR1as resulted in a phenotype similar to that of miR-7 knockdown, while injection of the miR-7 precursor partially reversed this phenotype, further implicating CDR1as as an antagonist of miR-7 [14]. The results of this study provided evidence that circRNAs may function as regulators of gene expression at the post-transcriptional level. In addition to these studies, Jeck *et al.* recently discovered over 25,000 circRNAs in human fibroblasts [187]. Due to the lack of exposed 5′ and 3′ ends, circRNAs are predicted to be resistant to degradation by cellular enzymes such as ribonucleases and thus more stable than linear RNAs. The increased stability of circRNAs makes these novel RNA molecules act as efficient miRNA sponges in modulating gene expression.

## 5. Conclusions

NcRNAs have gained intensive and growing attention for their potential as both regulators and biomarkers of cancers in the past two decades. We are now witnessing the beginning of a new era of identifying key players and determining their underlying mechanisms during oncogenesis. In this review, we are only able to summarize some studies of miRNAs, lncRNAs and circRNAs. We did not intentionally ignore other excellent reports, but just could not include them due to the enormous amounts of ncRNA-related studies and the limitation of this article. Many review papers have summarized the roles of ncRNAs in different diseases including cancers and some of them also discussed several other types of ncRNAs, such as snoRNA and small nuclear RNAs (snRNA).

Currently, many thousands of ncRNAs have been identified and their differential expression profiles in a variety of cancers or between normal and tumorigenic specimens have been demonstrated; however, only a small number of ncRNAs, especially lncRNAs, have been well characterized. Thus, what we have discovered is just a tip of the iceberg in this area. Understanding the functions and regulatory mechanisms of ncRNAs in cancer pathogenesis remains a fertile research field to be explored at least in next decade. Future studies are needed to dissect the upstream mechanisms regulating ncRNA expression and processing, and the downstream proteins or pathways mediated by ncRNAs at different stages of cancer development. These efforts may lead to the discovery of entirely novel regulatory mechanisms or advance our understanding for the currently recognized signaling pathways. The achievement of these ncRNA studies in cancers will definitely provide insights into discovering new biomarkers for cancer diagnosis and prognosis, unraveling novel therapeutic targets, and developing unconventional therapeutic modalities to reduce cancer-related mortality.
